# Health and Habitability in the Solar Decathlon University Competitions: Statistical Quantification and Real Influence on Comfort Conditions

**DOI:** 10.3390/ijerph17165926

**Published:** 2020-08-14

**Authors:** Rafael Herrera-Limones, Antonio Millán-Jiménez, Álvaro López-Escamilla, Miguel Torres-García

**Affiliations:** 1 University Institute of Architecture and Construction Sciences, Superior Technical School of Architecture, University of Seville, Av. Reina Mercedes 2, 41012 Seville, Spain; herrera@us.es; 2 Faculty of Medicine, University of Seville, Avda. Sánchez Pizjuán, s/n, 41009 Seville, Spain; millan472@gmail.com; 3 Energy Engineering Department. Superior Technical School of Engineering, University of Seville, Camino de los Descubrimientos, s/n, 41092 Seville, Spain; migueltorres@us.es

**Keywords:** health, habitat, comfort, sustainability, medicine, architecture, competition, university, education

## Abstract

Medicine and architecture are disciplines with the main objectives of satisfying the fundamental needs of human beings: health, comfort, well-being, safety, and ensuring an acceptable quality of life in a sustainable habitat. In both areas of knowledge, the advances and the most innovative proposals in the fields of research and teaching are focused on transversal knowledge and the use of learning methods through problem solving (learning by doing). The student competitions called “Solar Decathlon” are focused on the development of these concepts, in which prototypes of sustainable and, as far as possible, healthy social housing are tested. In these university competitions, the design of energy-efficient and comfortable living environments that contribute to the health of the occupants are encouraged; however, the methodology for evaluating the “comfort conditions” stipulated in the competition rules considers only parameters that can be monitored by sensors. For this article, the prototypes presented by the “Solar Decathlon Team of the University of Seville” to the editions of said competition held in Latin America and Europe (in 2015 and 2019, respectively) are being studied. The present research starts from the fact that the unique consideration of measurable indices (such as temperature, humidity, etc.), is clearly insufficient when it comes to evaluating the real conditions of habitability and comfort that a domestic architectural space presents. For this reason, a theoretical–practical analysis is carried out by means of surveys, with the final objective of determining a methodology for evaluating comfort—complementary to that of the competition—which assesses other relevant issues and which, in short, takes into account the repercussion on people’s health. From our analysis, we conclude that at least these two methodologies should be used to evaluate comfort because they are individually considered incomplete in terms of the data provided by each one of them. The survey-based methodology provides complementary information on comfort and health that could be taken into account in future editions of Solar Decathlon.

## 1. Introduction: Comfort and Health

Throughout time, human health and habitat have maintained a close relationship, derived from their common objective: to achieve minimum levels of comfort that allow the presence of the human race on planet Earth, within certain objective and subjective ranges of what we could call “quality of life”.

However, from the outset, it is essential to discriminate between the terms comfort and health... both from the medical point of view and from the architectural perspective, as there are differences between the two concepts that, however, are shared by both disciplines. 

Resorting to a literary simile, when the Spanish poet Juan Ramón Jiménez (Nobel Prize for Literature in 1956) describes an equine in the following terms: “Platero is small, furry, soft; so soft on the outside that one would say everything is made of cotton, and has no bones. Only the jet-black mirrors in his eyes are as firm as two black glass beetles (...)” [1], he does this by depicting a donkey as a comfortable animal, in the linguistic sense of producing well-being and seeking calm and even pleasure, since one senses that it must be pleasant to caress it, to embrace it, to ride it... This is a description, applied to an animal, of comfort. 

Now, would it be healthy to live with Platero? In other words, would it be advisable to breathe the air expelled by the donkey through its throat, through its never cleaned teeth, in a closed room? Would not the possibilities of contagion, due to the external and internal parasites that this type of animal usually carries, be obvious? Or, would not there be an evident risk of suffering an asthmatic crisis by a child allergic to the animal’s hair, compromising even his own life? 

Therefore, if we apply this literary–veterinary reflection to the design of the spaces in which we live, work or walk (or to their material configuration), we will obtain an obvious fact that must be a premise when undertaking such a task: to make something healthy comfortable and vice versa. To do so, it is essential to deepen our knowledge of the physiology of human beings: of their correct functioning as “psycho-biological machines”, given that, as the end user of the architectural fact, they are the recipients of the creative and constructive effort of the environment in which man’s life unfolds: the habitat in its broadest sense. 

## 2. Medicine vs. Architecture/Health vs. Habitability/Human Well-Being vs. Architectural Comfort

### 2.1. Medicine vs. Architecture: The Old, the New and the Very New Relationship between Disciplines

Medicine and architecture have the human being as the ultimate goal of their raison for being. This affirmation, a meeting point between these two disciplines, does not prevent them from seeking, through different paths, how to satisfy the fundamental needs of humanity, such as health, disease prevention, safety, comfort or well-being... in short, to configure a sustainable and healthy habitat. 

From the architectural point of view, responding to a certain need to express the most artistic aspect of construction design, the issues related to comfort, to the correct environmental conditioning and, in short, to providing the end user with minimum levels of healthiness, have not been sufficiently valued. On the other hand, from the medical point of view, factors related to diseases in the immediate building environment are sometimes decontextualized, and the architectural conditions that surround patients are not sufficiently assessed, which in many cases are closely related to the healthy or unhealthy parameters detected in the patients.

However, despite all this, what is evident by all means is that the relationship between both disciplines has evolved; thus, as opposed to the traditional vision that links both fields (according to the aforementioned common concern regarding human health and comfort), it happens that in recent times, derived from the planetary climatic crisis and, more specifically, from the different economic crises that have occurred in the West... a new field of transdisciplinary action has been appearing that incorporates the paradigms of sustainability and recycling. This brings with it, in the architectural field, a reconsideration of the built heritage and the rehabilitation of what already exists, at a time “when it is no longer necessary to build much more” [2] and, in the health field, a reconsideration of the term “health”, which is more linked to emergency.

In this sense, one could say that in May 2020, when half the world is literally confined to their homes as a result of the COVID-19 pandemic, we have an unparalleled opportunity to reconsider the relationship between architecture and medicine, and even to advance a relationship between the two, which is no longer new, but is rather very new. This also poses (derived from the prolonged lockdown) both new housing uses, and a rethinking of minimum comfort levels, to prevent deterioration of people’s health after isolation.

In the same way that it forces us to redesign public spaces, since they are potential places of propagation of virus and infections, for what the design of these will have to be tied to is evaluation from a sanitary point of view. Even more so in situations of pandemics, these open spaces turn into the most advisable, from the point of view of social meetings, in order to avoid accumulations of persons in closed spaces [3].

However, on closer inspection, perhaps this trans-disciplinary relationship is not so new... given that historically there have always been links—sometimes unsuspected—between architecture and the management of epidemic crises. Thus, for example, the plague epidemics that marked the Middle Ages, smallpox in the 17th century and cholera in the 19th, associated urban planning with the existence of certain basic medical structures; later, tuberculosis led to a radical change in the way of understanding the human habitat, with new technologies in glass, concrete, or more specifically, the establishment of rounded joints between walls and the floor, to avoid the accumulation of dust, considered lethal for the sick [4,5,6,7]. Subsequently, during the first great contemporary pandemic, the Spanish flu, the importance of controlling the environment and the built environment emerged once again, which ended up taking shape in the “Athens Charter” [8], in the early 20th century, which established minimum parameters regarding light, ventilation, materials and designs, specifically to favor the healthiness of the urban centers of the time, and of architecture in general.

Returning to the current pandemic situation, and regarding the relationship between architectural typological advances and health requirements, two lines of implementation of emergency solutions could be pointed out: on the one hand, new typologies of emergency hospital architecture are being developed, for mass infectious diseases (see ultra-fast construction hospitals in China); on the other hand, a new ephemeral health architecture has appeared that is emerging to accommodate the sick, the dead, the homeless, etc., of quick assembly or is inflatable, inside other existing buildings. Both solutions of this last typology, the one that could be considered inadequate (bullrings, football stadiums, sambodromes, etc.) and others that are much more suitable (such as the use of exhibition halls, covered sports centers or large multi-functional buildings), are taken into account.

One of the most paradigmatic cases may well be IFEMA [9] which, in March 2020, hosted the largest field hospital in the world, with underground facilities capable of supplying oxygen to hundreds of ICUs, but which three months earlier had hosted the “World Climate Summit COP25 Chile-Madrid” [10], and which, only one month earlier, had hosted “ARCOmadrid”, one of the main contemporary art fairs on the international circuit [11,12], linking in an almost diabolical way CLIMATE CRISIS + ART + HEALTH.

### 2.2. Common Parameters of Architectural Comfort and Their Impact on the Well-Being and Health of Users: Architectural Configuration vs. Conditions of Comfort and Environmental Health

Each individual has a personal and particular conception of what health and comfort is, along with a broader, general and less precise one. Therefore, defining both concepts is not easy since both terms include aspects that escape objective quantification and are related to sensations. Thus, from the medical discipline, one could find descriptions such as “I feel good”, “I am perfectly fine” or “I am a healthy person”, when one asks about the state of health of a person. On the other hand, you will find expressions such as “I am comfortable here”, “how cozy and pleasant this place is”, etc., when you ask about the sensation of comfort.

For the World Health Organization (WHO), health is a state of complete physical, mental and social well-being and not merely the absence of disease or infirmity [13]. This concept, coined in 1948, logically does not take into consideration more modern aspects, such as molecular biology, which allows the detection of diseases or infections before they manifest or are felt by the affected person. This broad definition issued by the WHO can, therefore, also be questioned, even though it is the one most widely accepted by the scientific community. 

Therefore, health is a state that is neither constant nor uniform and that would even allow a gradation of the level of health that each one has at each moment of his life. It is even difficult to measure it accurately, but not to evaluate it in consensual terms: absence of illness, absence of harmful habits, sensation of physical and mental well-being. Marc Lalonde, Canadian Minister of Health, stated that the health of the population does not depend exclusively on the healthcare provided by the government and, on this basis, defined the elements that condition health: human biology, environment, lifestyle and health systems [14,15,16]. The most influential on health is lifestyle, determined by each individual, followed by the environment; accepting that human biology, for the sake of coherence, allows for the health of the individual, and health systems prevent disease and care for the sick. 

Regarding comfort or well-being, it is even more complicated to find a uniform and objective definition because, as has been pointed out, it involves personal sensory, subjective and psychological aspects that are difficult to quantify. For the Royal Academy of the Spanish Language (RAE), comfort is well-being or material comfort, and well-being is the set of things necessary to live well. These definitions refer to the immediate environment, to materials or things that influence or lead a person to live well and “comfortably”.

We could safely assume that comfort is included in the definition of health and not the other way around, because to be comfortable we must be healthy. The opposite is not possible and even occasionally, what we understand as comfort will not be healthy. 

If we analyze the inherent aspects of the person that condition them to define a comfortable space, we will find age, sex, race, psychological state, illnesses or disabilities, etc.; as opposed to others that depend on the environment, external to the person, at the moment in which we evaluate comfort: natural or artificial light, noise, silence, color, texture, humidity, temperature, etc.

For all the above reasons, this article finds it essential to consider the analyzed factors, which traditionally constitute the parameters of scientific comfort: temperature, humidity, lighting, air quality, etc. (in our case, focused on social housing), although these comfort conditions do not always directly affect the health of the user. However, it is commonly understood that optimum comfort conditions in buildings are responsible for beneficial direct consequences on human health [17]. At the opposite extreme, we usually consider certain situations of presumed temporary comfort [18] which, however, lead to serious health problems: from bad postural habits to drugs.

There is a whole series of physical conditioning factors not considered in the classic parameters of “scientific comfort” [19], such as networks, ionization, defects in the orientation of the building, etc.; along with others of a psychic nature [20,21,22] which are also not usually considered, such as: environmental noise, security, intimacy or addictions [23].

However, minimum environmental comfort conditions are required in domestic spaces [24], using strategies focused on sustainability and low energy consumption [25], to ensure certain minimum levels of health, which have an impact on an acceptable quality of life. 

These environmental comfort parameters are, therefore, directly related to different pathologies, so that spaces designed specifically to maintain adequate levels of temperature, relative humidity and lighting can help both alleviate and prevent diseases.

The lack of such minimum levels is evident in cases of social housing, where the quality of their interior spaces, in a high percentage, does not meet minimum quality standards, with the consequences being felt by people with fewer resources, elderly and/or those with some type of illness [26].

In this sense, in relation to the health problems caused by this fact, a direct relationship has been detected between coronary heart diseases and spaces with low temperatures [27], and it has been shown that mortality from this type of illness increases in the winter months in countries where homes are not adequately equipped with thermal insulation. This situation, therefore, is aggravated in countries with hot climates [28], as a result of lower investment in the insulation of housing, and more specifically, in the case of social housing, so that we can relate energy poverty [29] with the increase in mortality from these diseases in the poorest sectors of society [30].

In the same way, you must take into account that very high and very low levels of air humidity in buildings, either directly or through biological or chemical agents, are harmful to health and can lead to acute and, more commonly, chronic diseases and, in extreme cases, deaths.

In this sense, relative humidity conditions in indoor spaces are strongly related to respiratory problems such as asthma [31,32,33], since, although high humidity is not considered to have a direct influence on this fact, the presence of mold does [34], which is sometimes a consequence of high humidity and poor air quality in the rooms [35,36,37].

As for sunlight, human beings have made use of its healing properties throughout history. Since the 19th century, the benefit of sunlight as a medicinal method in tuberculosis patients has been linked [38]. In fact, until well into the 19th century, no drugs were available for the treatment of this disease, so until then, treatment was carried out in buildings erected specifically for this purpose, which were located in mountainous areas or near the sea where the patient had sufficient exposure to sunlight and was in contact with uncontaminated air. These buildings were always oriented to the south or southeast to allow for “heliotherapy” or healing by sunlight [39]. It can, therefore, be said that medicine was architecture itself.

Nocturnal secretion of melatonin is also strongly linked to exposure to natural light; this hormone being related to diseases such as cancer, insomnia, depression, dementia, hypertension and diabetes, as well as sleep–wake regulation [40]. Especially relevant in this sense, according to the scientific literature, is the incidence in the elderly [41], among whom it has been shown that exposure to daylight is positively associated with urinary excretion of 6-sulfatoxymelatonin [42].

Therefore, today we are aware of the impact of natural light on people [43], and of its many benefits. Among them, natural lighting prevents the development of bacteria that cause respiratory effects and that find the best conditions for reproduction in the dark. It also strengthens our immune system and improves the cognitive function of our brain. Through natural light we can also obtain a feeling of well-being and comfort, which is directly related to a decrease in stress and fatigue. 

However, the benefits that architecture can bring to people’s health are not only related to quantifiable parameters. In research carried out in hospitals, with patients undergoing medical treatment, it has been shown that the environment, the decoration or the views that the patient has from the window of his or her room, have an influence both on reducing the recovery time and the lesser amount of medication that he or she needs, when these views are of landscaped areas rather than degraded areas [44]. In the same way, interior spaces with optimal hygrothermal conditions, in addition to a design that has spaces for patient distraction or garden areas, has a positive influence on patient recovery [45,46].

Another aspect related to the comfort of the inhabitant has to do with the materiality and the psychic impact that it has on the human being. Starting from some surveys, it has been possible to corroborate that wooden structures provide a greater sensation of comfort to the individual, as opposed to those made of concrete [47]. This, taking into account the subjectivity of comfort in each person, becomes a variable to be taken into account in the design and finishes of domestic spaces.

Thus, the aim of this article is to compare different comfort assessment methodologies to determine which is the most suitable from a health point of view. To this end, we will use the Solar Decathlon university competition as a case study, focusing on the social housing prototypes presented by the University of Seville in two recent editions of the competition.

## 3. Case Study: Solar Decathlon Competition

### 3.1. Solar Decathlon University Competition

The Solar Decathlon (SD) competition is the most prestigious international university competition on sustainable habitat, originally sponsored by the United States Department of Energy. Universities from all over the world participate in this competition in collaboration with institutions and companies with the aim of designing, building on a real scale and monitoring a prototype of a housing cell with the highest level of self-sufficiency and use of renewable energies.

During the creative and competitive process, undergraduate, master’s or doctoral students, tutored by teachers and researchers, develop ten tests (Table 1)—hence the name decathlon—apparently as diverse as: Sustainability, Innovation, Communication, Housing Operation, Social Relevance, Architecture, Engineering and Construction, Energy Balance, Energy Efficiency and Comfort Conditions, in a clear application of the so-called learning by doing methods [48,49,50].

The last of the ten reviewed tests, Comfort Conditions, is a clear opportunity to test the health conditions in the living environment; therefore, in the conviction that both in the medical and architectural disciplines, the advances and the most innovative proposals in the teaching and research fields aim at transdisciplinarity, this type of student competition for sustainable habitat, in which, as has been indicated, sustainable and—as far as possible—healthy social housing prototypes are tested, is extremely valid.

This converges with one of the trends with the longest tradition in educational innovation, the so-called integrated curriculum [51], that is, overcoming the division into academic disciplines in order to better respond to scientific progress [52].

Furthermore, it should be noted that these types of “university competitive tools”, the SD competitions, constitute a great opportunity to deepen the UNESCO SDGs for 2030, and given that the search for models of living is the original purpose of this line of research, the final aim is to minimize the environmental impact (in terms of convergence towards H2020) and to improve public health.

### 3.2. Participation of the University of Seville in Solar Decathlon

In hindsight, the first participation of the University of Seville in this teaching and research initiative dates back to 2010 (Table 2), in the first edition of the Solar Decathlon competition in Europe, and it will again take part in the 2012 edition (both held in Madrid). In 2015, the team was reformulated and the Aura Project was born, and a proposal was submitted to the first edition of the competition in Latin America (SDLAC 2015) [53]. In this edition, the Aura Prototype, in addition to being recognized with the 3rd Absolute Prize, won 1st Prize in the Comfort Conditions test, as well as several mentions and prizes in other categories [54].

Recently, in June 2019, the Solar Decathlon Team of the University of Seville starred in its last competition, which took place in the European edition held in Hungary, repeating the 1st Prize in the Comfort competition (and winning, among others, the prize in the Sustainability category).

This article focuses specifically on the participation of the Solar Decathlon Team of the University of Seville in the editions held in Colombia (in December 2015), and in Hungary (in June 2019), in which the issues already mentioned regarding the integrated curriculum were especially contemplated, in such a way that the process of the competitions, in general, and the design of the sustainable prototypes, in particular, was approached with a holistic approach, eliminating the barriers between disciplines and establishing a close collaboration between the different teaching and research centers of the University of Seville [55], especially highlighting the role of the Faculty of Medicine and the School of Architecture.

In order to properly implement the conditioning strategies in the AURA 1.0 prototype (in tropical climate) and in the AURA 3.1 prototype (in Mediterranean continental climate), it was logically necessary, first of all, to analyze what the comfort conditions were and under which ranges the competition was scoring the test [56,57], so that it could be adapted to the times of day, and under which strategies the prototype was scoring well and when it was not.

### 3.3. Solar Decathlon Latin America and the Caribbean 2015: AURA 1.0

At the design level, it is worth highlighting the objective of providing additional values to the minimum housing module, through conditioning strategies, contributing, on the one hand, to the physical health of the inhabitant by achieving optimum hygrothermal and lighting values; and on the other hand, to the psychic health, integrating “the street”, urbanity, territory, inside the house, since in this case we are dealing with a society very accustomed to interaction with the environment, so suppressing this component of the housing cell would cause a sensation of disconcert of the individual that could lead to pathology.

The prototype incorporated a series of reconfigurable elements that gave it a certain flexibility and meant that the house could be used in different situations and according to different family needs.

A multifunctional room with a movable partition, which can be made independent, as an additional bedroom, or kept fully open and attached to the living room, in the center of the home, depending on the people and time of day this space is used in the house. This mobile partition could be used not only to create a spatial relationship between the living room, the terrace and this multifunctional room, but also to achieve high levels of cross ventilation throughout the house. 

**Figure 1:**
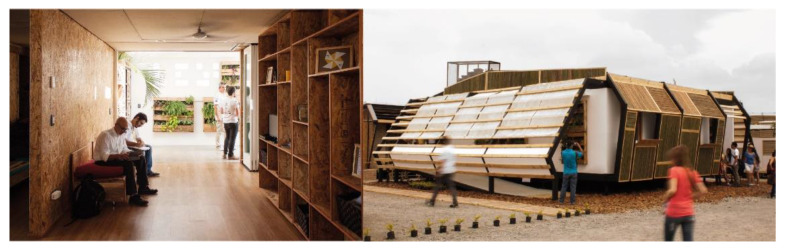
Photograph of the entrance hall seen from inside and South façade/Aura 1.0 prototype.

The outer skin can be opened for better natural interior lighting when needed, as well as to improve natural ventilation or simply to extend the view to the outside. The natural position of this skin, however, was to be closed to prevent the accumulation of dust and dirt outside the house without the need to remove the inescapable ventilation (Figure 1).

### 3.4. Solar Decathlon Europe 2019: AURA 3.1

The main objective of the project is the urban regeneration of residential neighborhoods in the European climate–energy context of the Mediterranean area. Solving hygrothermal comfort in residential spaces during the hot period of the year is a priority issue from the point of view of sustainability in a future scenario of climate change.

**Figure 2:**
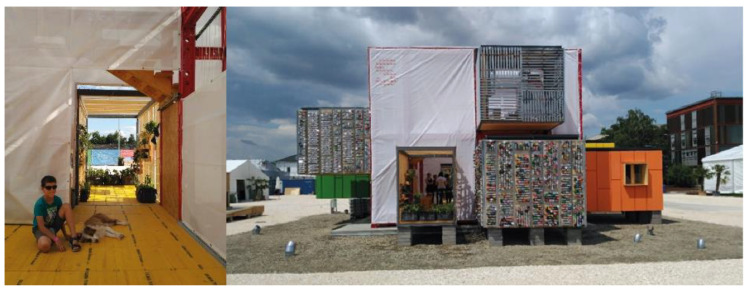
Interior photograph and a view from façade/Aura Prototype 3.1.

The strategy of Project Aura 3.1 (Figure 2) is based on a flexible, evolutionary and transformable construction system. The system is applicable, with infinite configurations, to rehabilitation projects of existing residential neighborhoods, preferably considering criteria of sustainability and efficiency, aimed at obtaining the maximum level of self-sufficiency through the use of renewable energy.

Monitoring of the prototype pavilion during the competition in Szentendre (Hungary) from 15 July 2019 to 24 July 2019 was the opportunity to obtain quantitative data on the validity of the Aura strategy. During these days, the prototype pavilion becomes a real urban laboratory where the qualities and performance of the system can be tested in a situation that is reasonably close to a real case of retrofitting social housing.

The strategy of controlled exchange with the outside made it possible to guarantee the best indoor comfort conditions in the tests with continuous measurements, obtaining the maximum score in indoor air quality and humidity and the second best score in indoor temperature in the competition period.

## 4. Comfort vs. Health: From the Theory of the Prototypes for the Solar Decathlon Competitions and the Objectivity of the Measurable Data, to the Reality of the Human Team. Methodologies

As indicated, during the creative and competitive process, ten tests are developed, among which are Sustainability, Innovation, Housing Operation, Social Relevance, Energy Efficiency and Comfort Conditions, the latter being a clear opportunity to test the health conditions of both the prototype pavilion with which it competes and its immediate living environment.

The concern of the members of the Solar Decathlon Team of the University of Seville, regarding the comfort conditions and the degree of healthiness provided by the designed models, is evident if we look at the results obtained in the two most recent editions in which they have competed: SDLatinoAmerica 2015 and SDEuropa 2019, given that in both they have won the 1st Prize in that category.

**Figure 3:**
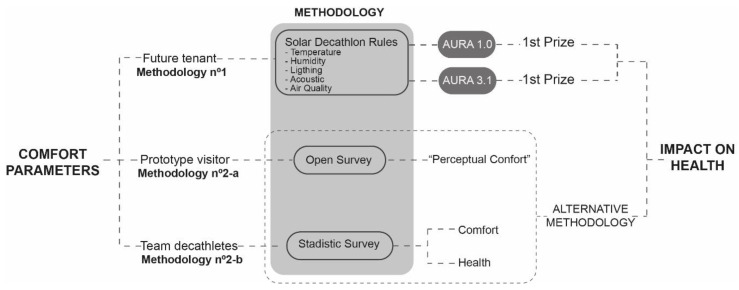
Methodology.

However, the main sense of this research is to go beyond the analysis of theoretical comfort parameters (temperature, humidity, air quality, lighting and acoustic conditions), which were scientifically measured during the competition. The aim is to compare different comfort evaluation methodologies from the point of view of the habitat and to determine which of them allows us to obtain data about the user’s comfort and health. In this sense, two methodologies are analyzed, using different profiles of inhabitants for their evaluation (Figure 3).

- Methodology no. 1: this is the one used by the Solar Decathlon competition organizers, and is based exclusively on measurable parameters: temperature, relative humidity, natural lighting and outside noise. It is an objective methodology, focused on satisfying the comfort of the future inhabitants of the prototypes presented in the competition. Under this methodology, the University of Seville won first prize in the 2015 (Cali, Colombia) and 2019 (Budapest, Hungary) editions of Solar Decathlon. However, this research defends that the comfort of the habitat should not be exclusively quantifiable and objective, but it is also fundamental to know what that living space transmits and how it makes the people who interact with it feel. Therefore, because methodology no. 1 is considered incomplete from the point of view of the analysis of comfort on the user’s health, the members of the University of Seville carried out two other alternative procedures that were tested in the Solar Decathlon 2015 (methodology no. 2-a) and 2019 (methodology no. 2-b).
- Methodology no. 2: based on conducting a survey of visitors to the prototype and participants.
  1. Visitor surveys: an open qualitative survey is conducted, taking as a study profile the people who visited the prototype during the 10 days it was on display and open to the public. This type of survey was chosen because it was aimed at people without technical qualifications but who, hypothetically, might one day inhabit that dwelling and were visiting it at that time. Therefore, for this research, the aim is not to focus on any specific aspect of the public’s view, but rather to learn to see the dwelling from another perspective. The aim was to know the sensations that the house provoked in the public, e.g., pleasant, happy, depressive, sad and confused feelings.
  2. Survey of participants: just as methodology no. 1 analyzed the comfort conditions of the future tenants of the dwelling and the visitor’s survey analyzed the impact of the prototype on visitors, this one aims to evaluate the comfort and its influence on the health of the members of the University of Seville team who participated in the assembly, exhibition and dismantling of the prototype across, in total, approximately 30 days. The aim is to test a type of survey whose objective is to relate the comfort and health of the participants in a direct manner, that is to say, the Solar Decathlon experience is used to test this methodology. This methodology has subsequently been validated by the Clinical Research Ethics Committee of the Nuestra Señora de Valme University Hospital in Seville (Spain), and a broader version is being used to evaluate the impact of confinement due to COVID-19 on architecture and medical students at the University of Seville.

### 4.1. Methodology No. 1: Evaluation of Comfort Conditions at Solar Decathlon

The SD competition assumes that comfort is not uniquely measurable and that this assessment must be divided into the parameters: temperature, humidity, acoustics, air quality and lighting. These are parameters that make it possible, objectively, to determine whether the environment created inside the prototype is “comfortable” by means of a score ranking. However, are these parameters the only ones that make it possible to assess comfort or, even more importantly, to assess how healthy the prototype is?

No, as argued above, such comfort assessment in SD is, in our view, incomplete or biased towards aspects external to the human being. There is, therefore, a deficit in the assessment of the prototype in terms of its contribution to the health of its future occupant, and the assessment of physical (temperature, light, humidity, noise) or chemical (CO_2_) aspects is limited. 

Table 3 shows the criteria under which comfort were evaluated at Solar Decathlon Latin America 2015. In the European edition of 2019, the criteria were coincidental, introducing a slight variation in the range of temperatures considered in the comfort range, including, in addition, the measurement of air quality through CO_2_.

However, there is also another paradox related to the design process and the operating phase of the prototypes during the competition period: the assessment of the various pavilions would be aimed at the theoretical tenants of the sustainable social housing that the prototypes represent. However, as they are being made in real time during the course of the competition, they are actually affecting the visitors who enter them: the general public, the members of the jury and, ultimately, the members of the teams that carry out the assembly and then remain in the Solar Village during the course of the competition.

### 4.2. Methodology No. 2-a: Evaluation of Visitor Perception: SDLAC 2015

As stated above, and despite the “objective” data obtained by the measurable parameters that are part of the Comfort Conditions Test, the team’s interest during the competitive phase of the Solar Decathlon Latin America and Caribbean 2015 held in Cali (Colombia) does not disregard the “non-measurable” parameters that, as we have pointed out, also influence the individual’s comfort. For this reason, during the days of the competition, in which the house was exposed to the public, a series of surveys were carried out on the people who came to see the AURA 1.0 house in the Solar Village (Cali, Colombia) about the comfort they had felt inside, not only from the hygrothermal point of view, but also from the mental point of view, deepening the sensations they had had in relation to the materiality, decoration, smell, size, distribution, spatiality, etc.

Thus, when we talk about comfort conditions, we do not only refer to measurable parameters, but we also recognize that there is a component based on the sensations that space transmits to the individual, which causes comfort or discomfort.

**Figure 4:**
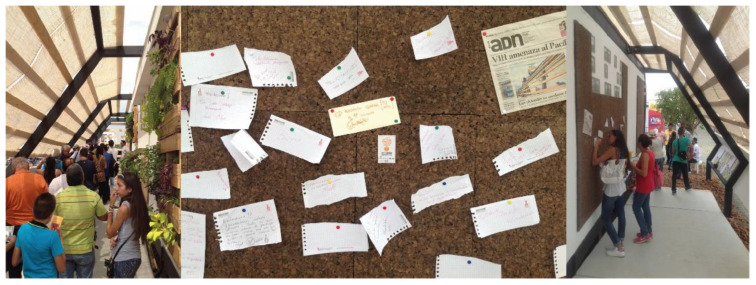
Survey integrated into the exhibition tour.

Therefore, part of the experience of visiting this house was to participate in a survey contextualized in the route of the exhibition prototype (Figure 4). This forms part of the methodology derived from a perception study: the project team was not interested, now, in knowing the opinion on the technical–architectural aspects seen by the visitor, given that they would not be experts on the subject (just as the hypothetical inhabitants of this prototypical social dwelling would not be). 

It is, therefore, an “open-ended qualitative survey”, the only generic question being based on the aspects that visitors highlighted from the prototype. This methodology gives the respondent the freedom to highlight non-technical aspects that in a closed survey, and elaborated by technicians, do not exist.

### 4.3. Methodology No. 2-b: Evaluation of the Comfort of the Participants in the Competition: SDE 2019

In the European edition of the Solar Decathlon in 2019, the SD-US Team considers (in the same sense as the Latin edition in 2015, but with the desire to take a further step in thinking about comfort and health) testing the state of health and comfort experienced by the members of the SD-US Team, both during the assembly of the prototype and during the scoring days of the various tests. 

**Figure 5:**
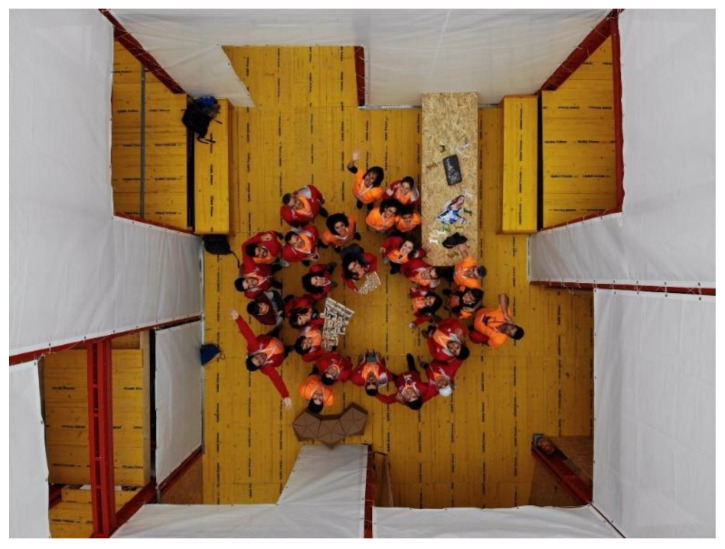
Survey.

This means attending or, rather, focusing not on the theoretical inhabitants of the sustainable social housing represented by the prototype; nor does it mean attending the event public but, in short, knowing the state of health of the decathletes, teachers and researchers of the team, and knowing their assessment of the comfort conditions they enjoyed both in the Solar Village and in their resting places, during that period (Figure 5).

As already mentioned, this research process involves going beyond the mere measurable tests of the Solar Decathlon Competition (specifically the Comfort Conditions Test), to find out about the health and comfort conditions of the members of the SD Team, both in the Aura 3.1 prototype itself, during its construction, and in the facilities of the Solar Village located in Szentendre (near Budapest), and even to sound out the conditions that surrounded their stay during that period of time (rest, leisure, etc.).

The design of the survey took into account two major aspects: one related to health and the other to comfort. It is an anonymous survey carried out between 10 and 15 July of the 30 members of the Aura team of the University of Seville who have participated with a physical presence in the Solar Village in one of the phases of the Solar Decathlon Europe competition. A survey was designed and transformed into a questionnaire using the Google Forms^®^ tool on the Google^®^ internet platform.

The questionnaire consists of 45 questions with multiple choice answers, free text and a numerical scale. 

All participants were informed of the nature of the study and consented to participate by providing their email, exclusively, for the submission of the form. Anonymity was guaranteed throughout the data collection and processing process.

The following personal data were requested: date of birth, sex, weight, height, previous or current illnesses and consumption of toxic substances. The Body Mass Index (BMI) was calculated to establish the degree of overweight and obesity according to World Health Organization (WHO) standards. In order to know the alterations in health during their stay, they were asked about neurological, digestive, traumatic or articular, dermatological, respiratory and infectious pathologies (Table 4).

The comfort of the decathletes and the rest of the members of the SD-US Team, was valued in the following aspects (Table 5):

- Rest: quality of sleep, nightly noise, rest area
- Social relations: with your own team and with the rest of the competitors
- Possibility of leisure activities
- Toilet quality
- Food quality and dining area
- Waste recycling
- Healthcare and care point for the organization and work area at Solar Village

For the analysis of the data, the IBM^®^ SPSSS^®^ Statistics version 25 for Macintosh, University of Seville license, was used. The quantitative variables are expressed using the usual statistics: frequency, mean, median, minimum and maximum. The qualitative variables are expressed by means of absolute frequencies and percentages.

## 5. Results and Discussion

### 5.1. Survey Results to Assess the Visitors’ Perceptions

**Figure 6:**
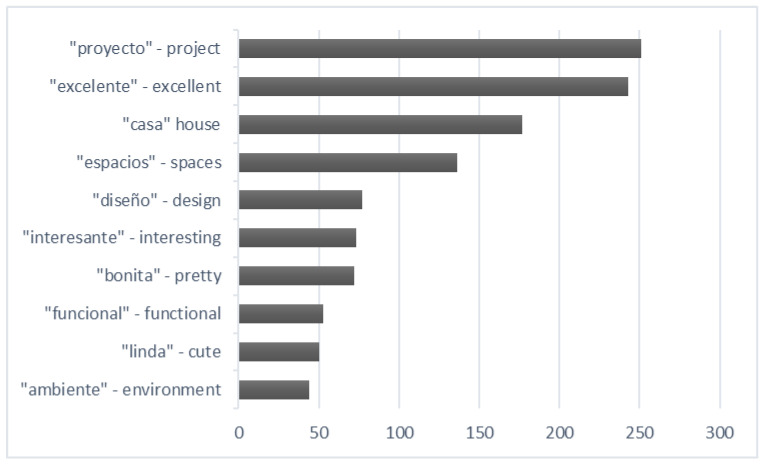
Terms most used by respondents.

**Figure 7:**
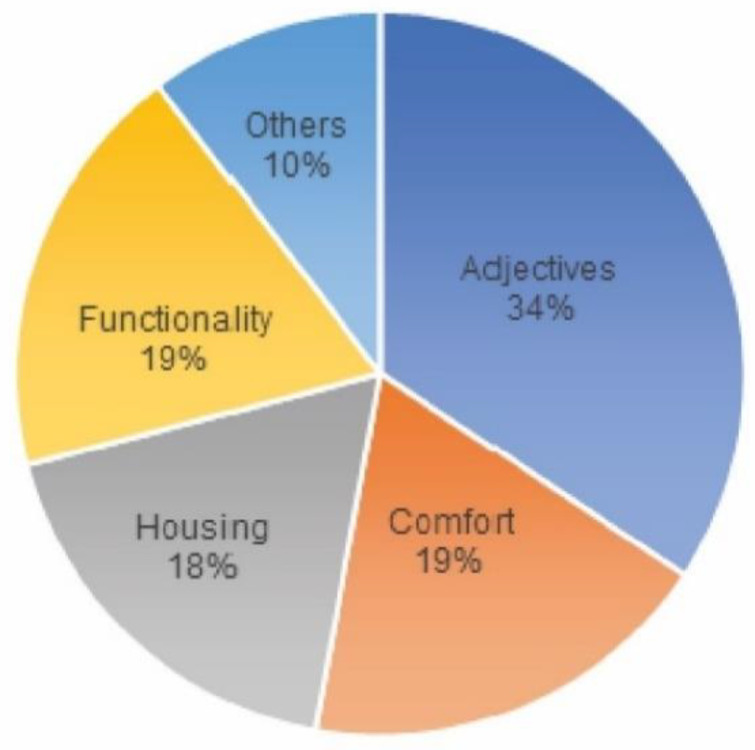
Highlights by respondents.

The survey carried out at the Solar Decathlon Latin American in Colombia in December 2015 was converted into a quantitative survey through language, this is, based on the participation of 1312 visitors, a study of the concepts that mostly emphasize the perceptions of visitors (Figure 6). Subsequently, they were grouped by topics on what stood out in each survey (Figure 7), without these being predefined or asked to visitors: the aforementioned gives us a vision of what the dwelling perceptively transmits.

From the thematic classification we made of the concepts, we can deduce that 34% of the comments used at least one of the following terms: “excellent”, “beautiful”, “pretty” and “spectacular”. On the other hand, 19% referred to comfort, as well as the same percentage expressed about the functionality of the prototype. It should also be noted that 18% of the visitors’ comments used at least the word “house” or “dwelling”, which makes us understand that the prototype was in line with the concept that society has interiorized as a living space.

As for comfort, the main words used by the visitors to define the prototype as such were (from more to less frequently): atmosphere, cool, comfortable, materials, pleasant and ventilation. The atmosphere created in a house, in the positive sense of the definition, makes the visitor feel good, does not generate rejection and is free from noise, olfactory or visual pollution. In the temperature and humidity conditions in which SD15 was developed, discovering a fresh and ventilated place is a reason for comfort and well-being. The extensive review carried out by Monika Frontczak et al. [58] showed that the interior temperature is the most important factor related to the sensation of comfort. However, thermal perception does not depend exclusively on the subject’s neurological record. Thermal and comfort perception is influenced by physical condition which, in turn, depends on sex, age, body fat, mood and adaptability through thermogenesis or evaporation [59]. Therefore, the relationship between environment and host is bidirectional: the physical conditions of temperature, humidity, light, ventilation, materials or sound influence the visitor, but their subjective perception is more complex than the physical or chemical parameters that surround them. Hence, the diversity of words used to attempt to define the prototype as comfortable. 

### 5.2. Results of the Survey to Evaluate the Comfort of the Participants in the Competition

**Figure 8:**
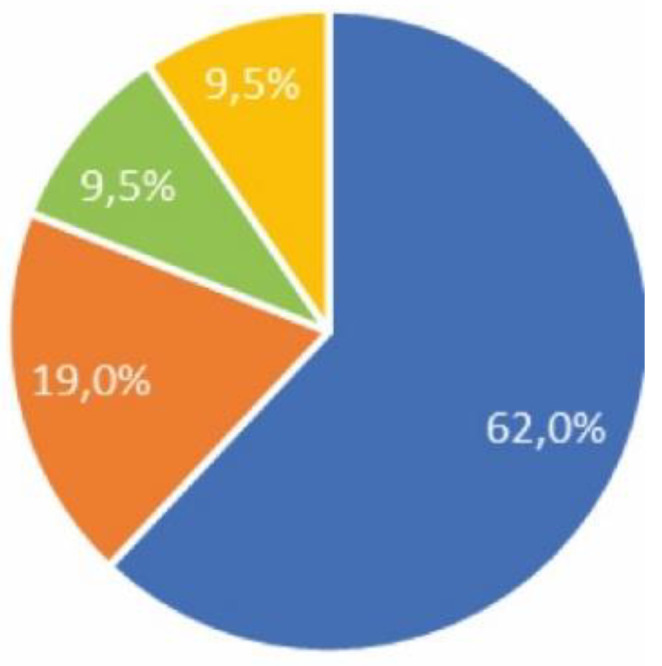
Health survey.

There was a response from 21 of the 30 respondents (70%); of these 57.1% were men, 33.3% women and 9.5% preferred not to say (Figure 8). The average age was 34 years with a median of 25 years and a range of 19 to 56 years. The average stay in Solar Village was 13.1 days with a median of 11 and a range of 4 to 30.

#### 5.2.1. Health Outcomes

62% reported being healthy, 19% food allergy, 9.5% respiratory allergy and 9.5% had undergone surgery (Figure 8). 

**Figure 9:**
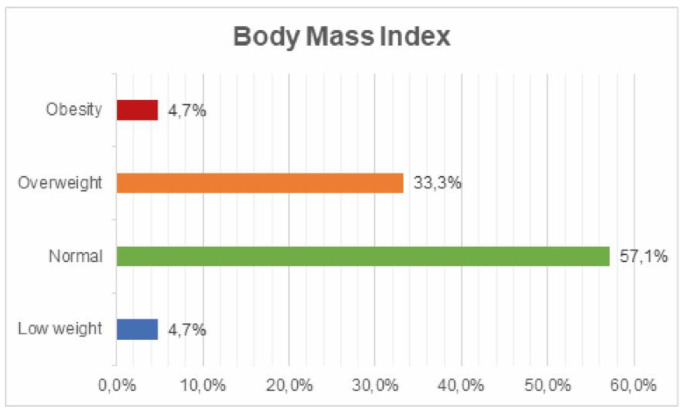
Body mass index value survey.

Classification according to body mass index value: 57.1% normal weight; 33.3% overweight; 4.7% obesity; 4.7% underweight (Figure 9). 

**Figure 10:**
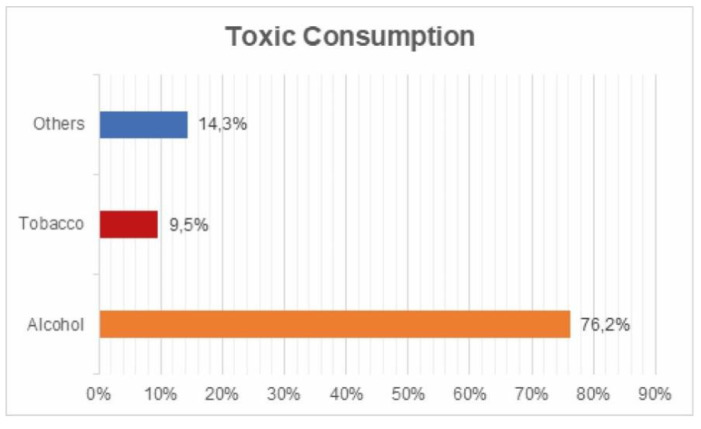
Toxic substances survey.

Consumption of toxic substances: 76.2% admitted consuming alcohol; 9.5% tobacco; 14.3% other substances (Figure 10).

**Figure 11:**
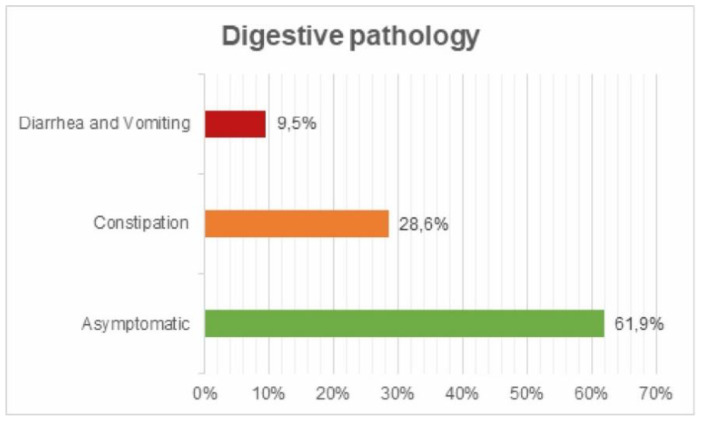
Digestive pathology survey.

Digestive pathology: 61.9% asymptomatic; 28.6% constipation; 9.5% diarrhea and vomiting (Figure 11).

No respiratory problems were detected.

**Figure 12:**
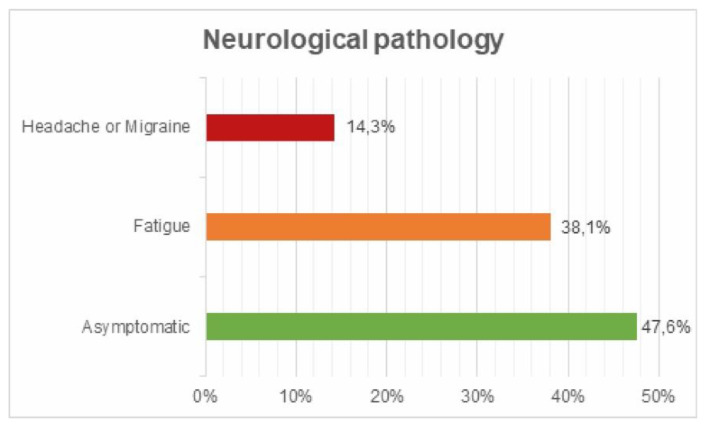
Neurological pathology survey.

Neurological pathology: 38.1% expressed tiredness or lack of rest and 14.3% headache or migraine (Figure 12).

**Figure 13:**
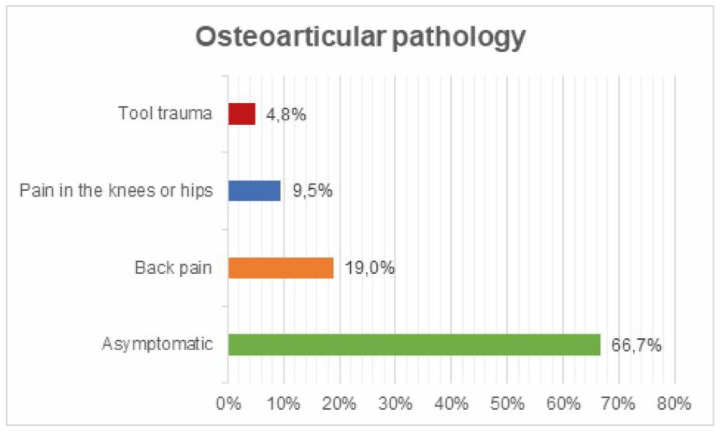
Osteoarticular pathology survey.

Osteoarticular pathology: 19% reported back pain, 9.5% knee or hip pain and 4.8% tool trauma. (Figure 13). 

Dermatological pathology: 85.7% suffered from mosquito bites. 

#### 5.2.2. Comfort Results

**Figure 14:**
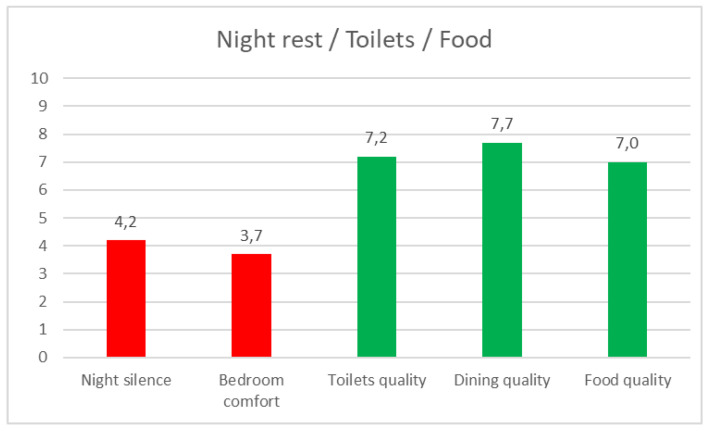
Night rest/Toilets/Food.

Night rest/Toilets/Food (Figure 14):

- 100% of the team members slept at the campsite.
- Silence during sleeping hours: 4.2 (0 much noise and 10 silence).
- Comfort of the night rest area: 3.7 (10 maximum comfort).
- Food quality: 7 out of 10.
- Frequency of consumption of ultra-processed or plastic-packed or industrially produced food: 6.1 (0 never and 10 daily).
- 28% found that alternative foods were available for people with allergies or intolerances.

**Figure 15:**
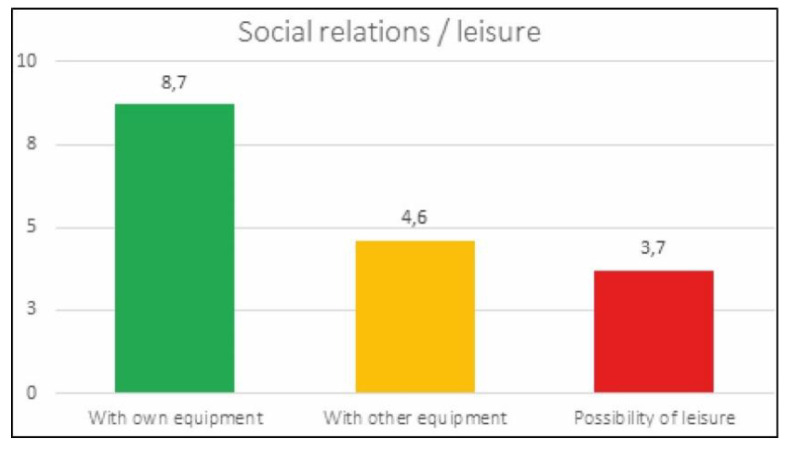
Social relations.

Social relations and possibility of leisure (Figure 15):

- With people from the team itself: 8.7 out of 10.
- Possibility of relationship with members of other teams: 4.6 out of 10.
- Possibilities of leisure activities: 3.7 out of 10.

Knowledge of the existence of a healthcare point: 15 out of 21 (71.4%). 

**Figure 16:**
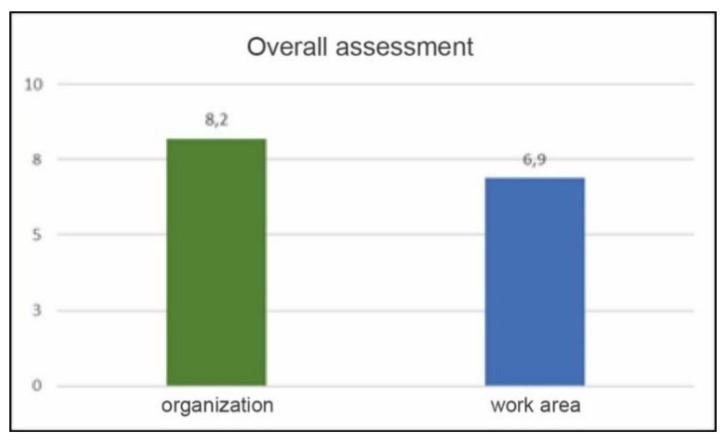
Overall assessment.

Overall assessment of the work area and attention by the organization (Figure 16):

- Attention by the organization: 8.2 out of 10.
- Assigned work area rating: 6.9 out of 10.

The health results in terms of overweight and obesity rates reflect quite accurately the values obtained in the epidemiological studies carried out in Spain [60,61]. However, an external observer would expect better results in this aspect, since it is a group of university teachers and students with a low average age and a high cultural level. Obesity and overweight have become the great epidemic of the 21st century affecting mainly western countries [62]. Perhaps in this aspect it is necessary to consider how architecture can help reduce the risk of obesity and if this is not possible, at least not increase it. To this end, the construction and planning of residential areas should have nearby open spaces, both for sport and public gardens, where aerobic exercise is not limited to the community’s courtyard. The access through comfortable stairs due to its design and the way that they are invited to be used through lightening, decoration, messages, etc. The prototype, as an isolated element of a building or urbanization, does not allow exploring these possibilities. 

The recognized consumption of toxic substances shows some worrying results, especially if we consider that it is a group of university students participating in a prestigious international competition. The consumption of alcohol by 76.2% of respondents stands out, which coincides with the data published in the report of the Spanish Observatory of Drugs and Addictions for the year 2019 [63]. This report states that 75.2% had consumed alcohol in the last 12 months, 62.7% in the last 30 days and 7.4% daily in the last 30 days. Addictions go against health and do not favor healthy habits, which would reflect a deficit in the awareness of the “constructors” of healthy prototypes [64,65]. 

The most frequent pathology, discarding the mosquito bites inherent to the place and date of the competition, back and joint pain, with an incidence of 28.5%, deserves some special attention. No participant reported any joint pathology or back pain prior to the competition phase, so these data could reflect either a poor prediction of “occupational risks” during the construction of the prototype or poor planning of night rest. Regarding the first option, it is possible that there is a direct correlation between the high theoretical level of the prototype designers, but a low practical level or expertise in its construction. This would lead to contractures due to lack of practice in certain efforts or prolonged execution times due to the need to shorten the assembly period, which is assessed by the organization. Both aspects could be taken into account in future editions. 

Regarding the second possibility, poor night rest, the rating expressed is quite significant: average score of comfort in the bedroom 3.7 out of 10, and of silence during the night rest period 4.2 out of 10. Both data reflect the difficulties faced by the participants in achieving a good night’s sleep. One of the aspects that could have caused such unfavorable conditions was the choice of the campsite as a resting place for all of them. In this environment neither the acoustic insulation, nor the temperature, nor the humidity, nor the level of carbon dioxide had to be adequate and, of course, they were not monitored. It is contradictory, or at least paradoxical, that the designers and builders of the prototype best valued for these parameters in the competition executed it in the opposite conditions. It is worth a reflection by the organizers of the event to guarantee, at least, some conditions of comfort in the night rest that reflect those with which the prototypes are judged. 

Regarding social relations during the competition, it is logical that between the members of the team these are strengthened or grow stimulated by the common objective of obtaining the best results. This is reflected by the respondents giving a score of 8.7 out of 10 when asked about it. However, it would be expected that an international competition among university students would promote the interrelationship between members of different teams and cultures, but this was not the case. In this way, leisure activities that support these relationships were not favored, rating 3.7 out of 10, nor were these relationships facilitated between members of different teams, rating 4.6 out of 10. Human beings are intrinsically social and the well-being of a person also depends on the quality and quantity of their social relationships [66]. It is enough to remember the suffering of those who are not accepted or directly rejected by the social environment. Favoring these social relations could also be integrated in the valuation of the prototype; taking into account common spaces to relate or where the presence of balconies–terraces allow communication, at least visually, between neighbors. 

The common spaces, toilets and dining room were valued with marks higher than 7 out of 10, reflecting an aspect well cared for by the organization. In the same way, the participants valued the attention of the organizers with a score of 8.2 out of 10. 

There is no doubt that food is one of the basic pillars of health; hence, social activities that generate comfort and well-being are developed around it. The quality of the food served in the dining room was rated positively with a score of 7 out of 10. However, the frequency of consumption of processed, ultra-processed or bottled food and drink was high during the days of the competition: 6.2 out of 10, with 10 being consumed daily and 0 not being consumed at all.

In this way, and after the analysis, we can say that the 2-a and 2-b methodologies allow us to obtain information about the sensations, comfort aspects, health of the visitors, and also of the students and teachers who participated in the prototype. 

The health survey, applied to a group of people, is the procedure of choice for finding out about subjective aspects linked to health: symptoms, personal habits, rest, exposure to toxins or risk situations, etc. It allows, therefore, the knowledge and monitoring of the state of health and also of the comfort perceived and expressed through the questions asked. It is a method that allows direct exploration of the person, his or her perception of health and comfort, and these data cannot be obtained from routine analytical records [67,68].

Both terms, comfort and health, are linked but not similar, and may even be opposite. Thus, resting on a soft structure or in an excessively cold or hot room may be comfortable, but not healthy. Furthermore, and vice versa, the consistency of a structure designed for healthy rest or the ideal temperature of a room may be healthy, but not comfortable for some people. The survey, as a tool to explore personal, subjective sensations and specific aspects related to health and habits, allows us to analyze and quantify how healthy and comfortable the participation was. 

The survey used, in the case of SD, has allowed us to have a new vision, and from another perspective, of the comfort and health linked to this competition.

## 6. Conclusions

The “Solar Decathlon” university sustainable habitat competitions encourage design in energy-efficient and comfortable environments that contribute to the health of the occupants. To this end, 10 tests have been established, of which Comfort Conditions is the most important. The evaluation system for this test is based exclusively on the consideration of monitorable data, obtained by means of sensors located inside the prototypes.

In the present investigation, it is concluded that the sole consideration of mechanically/electronically measurable parameters is clearly insufficient when evaluating the real conditions of habitability and comfort presented by a domestic architectural space and that, of course, this does not represent an example of the measurement of people’s health: that is why many other personal variables should be incorporated in addition to those obtained from the measurement of the sensors.

This is why it is essential to undertake a theoretical–practical analysis of a wider spectrum and broad holistic nature, with the final objective of determining a “methodology for evaluating comfort, complementary to the data analysis” which assesses other relevant issues and even takes into account the final repercussion on people’s health (the public that visits the prototypes, or the decathletes themselves who build and live in them during the competitive phase).

Thus, the use of the survey as a tool for evaluating health and comfort acquires a real and practical value that, at present, is far beyond the purposes that the Solar Decathlon competitions set out in their rules; therefore, it would be desirable to incorporate them into the competitive process.

Complementarily, this work proposes that the evaluation be established in a broader way: considering, on the one hand, the visitors to the prototypes and, on the other, the members of the teams in the competition. 

As for the former, through a linguistic study we can find out what sensations the prototype transmits. It is worth noting that, in the AURA 1.0 project, 18% of those surveyed recognized the proposal as a “house” or “dwelling”, and that 19% referred to comfort. This means that a large percentage of those surveyed identified in the project the necessary conditions for optimum habitability.

With respect to the participants in the event (decathletes) Solar Decathlon Europe 2019, where the University of Seville presented the AURA 3.1 project, the results obtained reflect deficiencies in comfort conditions during their stay, especially in night rest, social relations and leisure. Similarly, the data on health show worrying deficiencies in the care of their own health (expressed in overweight and alcohol consumption). It is surprising, and even paradoxical, that these same athletes, who managed to design the winning prototype of the “comfort conditions” test, do not enjoy optimal comfort conditions during their stay, nor are they aware of taking care of their own health. 

From our analysis we conclude that, in order to evaluate comfort, at least the two methodological models presented in this publication should be used (indoor space monitoring and user surveys that complement these data), since both methodologies are complementary. In any case, for the purposes of future editions of the Solar Decathlon Competition (as well as in other competitive calls on sustainable habitat), it is proposed that the survey-based methodology be applicable, due to its complementary information on comfort and health.

Finally (and in relation to the generality of the built habitat), it could be proposed that the methodology proposed here, based on the direct survey of the opinion of the people who live in the architecture, could be extrapolated to any building that is built; and that is not usually done. In this way, a great opportunity to advance in the interconnection of the medical and architectural disciplines is lost. Furthermore, if the health and comfort conditions of the users were taken into account in future designs, the health and comfort of future generations would be improved. In these times of pandemic and confinement worldwide, this is even more essential.

## Figures and Tables

**Table 1:** Analysis of the 10 contests of the competition.

Test	Description
Architecture	Housing evaluation with architectural design.
Engineering and construction	A jury evaluates the coherence between the structural, electrical, plumbing and solar design, and its ability to be assembled in 10 days.
Energy efficiency	Through a monitoring system, an adequate design is evaluated to achieve a reduced energy consumption.
Sustainability	A jury evaluates whether the prototype meets the objective of long-term environmental impact reduction.
Communication and marketing	The marketing and communication strategies used by each participating team to generate social awareness are highlighted.
Urban design and accessibility	An urban design applicable to the Latin American and Caribbean context is promoted in order to achieve an innovative proposal based on the development of low-cost housing.
Innovation	Evaluate the implementation of solutions to improve the quality of life of home users.
Characteristics of the housing	Measures the efficiency and functionality of a set of applications to ensure normal operation of the home and reduced energy consumption.
Electricity balance	Measures the balance between energy generation and consumption.
Comfort conditions	Through sensors distributed throughout the house, comfort parameters are monitored: temperature, humidity, acoustics and lighting.

**Table 2:** Timeline of editions of the Solar Decathlon competition.

Solar Decathlon	Country	Year
USA	Washington DC, USA	2002
	Washington DC, USA	2005
	Washington DC, USA	2007
	Washington DC, USA	2009
	Washington DC, USA	2011
	Irvine, CA, USA	2013
	Irvine, CA, USA	2015
	Denver, Colorado	2017
Africa	Ben Guerir, Morocco	2019
China	Datong	2013
	Dezhou	2018
Europe	Madrid, Spain	2010
	Madrid, Spain	2012
	Paris–Versailles, France	2014
	Szentendre–Budapest, Hungary	2019—Case Study
	Wuppertal, Germany	2021
Latin America and Caribbean	Santiago de Cali, Colombia	2015—Case Study
	Santiago de Cali, Colombia	2019
Middle East	Dubai, United Arab Emirates	2018
	Dubai, United Arab Emirates	2020

**Table 3:** Comfort test subcategory score range in SDLAC 2015.

Temperature Tmin = 24 °C Tmax = 28 °C	Full points	Tmin ≤ Temperature ≤ Tmax
	Reduced points	Tmin − 2 ≤ Temperature ≤ Tmax
		Tmin ≤ Temperature ≤ Tmax + 2
	No points	Tmax + 2 ≤ Temperature ≤ Tmin − 2
Relative humidity	Full points	Relative humidity ≤ 60%
	Reduced points	60% < Relative humidity < 70%
	No points	Relative humidity ≥ 70%
Natural lighting	Full points	4% < Daylight factor
	Reduced points	2.5% < Daylight factor < 4%
	No points	Daylight factor < 2.5%
Acoustic value	Full points	Acoustic value ≥ 42 dB
	Reduced points	30 dB < Acoustic value < 42 dB
	No points	Acoustic value < 30 dB
Reverberation time	Full points	Reverberation time ≤ 0.8 s
	Reduced points	0.8 s < Reverberation time < 1.2 s
	No points	Reverberation time > 1.2 s

**Table 4:** Health survey.

1. Birthdate	
2. Sex	
3. Weight	
4. Height	
5. Clinical history	a. Respiratory allergy
	b. Food allergy
	c. Any chronic disease
	d. I receive medical treatment
	e. I have been surgically operated
	f. I’m healthy
6. Consume of any of the following substances	a. Alcohol
	b. Tobacco
	c. Marijuana
	d. Hashish
	e. Others
7. Digestive problems during your stay	a. Diarrhea
	b. Constipation
	c. Abdominal pain without diarrhea or vomiting
	d. Vomiting
	e. Pyrosis
	f. I was perfectly at the digestive level
8. Respiratory problems during your stay	a. Cough
	b. Respiratory difficulty (such as asthma, whistles, etc.)
	c. I was great at the respiratory level
9. Neurological problems during your stay	a. Headache or migraine
	b. Motor deficit (loss of strength)
	c. Sensory deficit (loss of sensibility)
	d. Loss of vision
	e. Insomnia problems
	f. Feeling of exhaustion or lack of sufficient rest
10. Joint or bone problems during your stay	a. Bone fracture
	b. Ankle sprain
	c. Wrist sprain
	d. Contusion (blows by fall)
	e. Trauma with a tool (hammering somewhere, hit with structure…)
11. Skin or mucous lesions	a. Sun burn
	b. Burn by some instruments or hot utensil with flame
	c. Hives or allergies on the skin (hives, redness, itching…)
	d. Insect bite (describe later if mosquitoes, spiders, wasp…)
	e. Lesion type abrasion or abrasion by rubbing when falling
	f. Cuts in the skin
	g. Conjunctivitis or lesions on the lips or in the mucosa of the mouth
12. Infectious processes	a. Pharyngitis or tonsillitis
	b. Otitis
	c. Pneumonia
	d. Diarrhea
	e. Urinary infection
	f. Skin infection
	g. Infection in genital area
	h. Dental problems (pain, phlegmon, etc.)

**Table 5:** Comfort survey

1. Describe where you slept	Hotel
	Camping
	House
	Other
2. General comfort of your place of rest (scale 0 to 10): 0 if was not comfortable 10 if it was very comfortable	
3. Noise during sleep hours (scale 0 to 10) 0 if very annoying 10 if there was no noise	
4. The toilets in solar village were clean and well-kept (scale 0 to 10) 0 if they were dirty and/or careless 10 if they were kept perfectly	
5. During your stay, you have separated the remains of organic matter from packaging, glass, cardboard, other (scale 0 to 10) 0 if never 10 if always	
6. The dining area was adequate in Solar Village? (scale 0 to 10) 0 if not good enough to eat 10 if it was perfect to eat	
7. The food in solar village was (scale 0 to 10) 0 if it was very bad quality 10 if it was healthy and appetizing	
8. You have consumed food/drink packed in plastic: soft drinks, ultra-processed food, industrial pastry, etc. (scale 0 to 10) 0 never 10 daily	
9. Were there different options of meals for allergic people, intolerances or other food preferences?	Yes
	Not
	I do not know
10. How do you consider transportation from the resting place to solar (scale 0 to 10) 0 was very bad 10 was ideal	
11. Leisure activities or recreational activities 0 if not possibility of leisure 10 if there were many leisure options	
12. How was your relationship with your colleages? (scale 0 to 10) 0 bad 10 very good	
13. Activities have been facilitated to interact with members of other teams? 0 any 10 many and varied	
14. How do you consider working area in Solar Village (scale 0 to 10) 0 if very poor 10 if perfect to work	
15. How do you consider resting area in the Solar (scale 0 to 10) 0 non-existent 10 perfect to rest	
16. How does the staff attend you and your team? (scale 0 to 10) 0 if it felt fatal treated 10 if it felt great attended	
